# Supplementary material for: Particle interactions and their effect on magnetic particle spectroscopy and imaging
Source: Nanoscale. 2022 Mar 22;14(19):7163–73. doi: 10.1039/d1nr08402j (PMC9119029; doi:10.1039/d1nr08402j)
Supplement: NR-014-D1NR08402J-s001 [file NR-014-D1NR08402J-s001.pdf]

Electronic Supplementary Material (ESI) for Nanoscale.
This journal is © The Royal Society of Chemistry 2022

Particle interactions and their effect on magnetic particle spectroscopy and imaging

Supporting Information

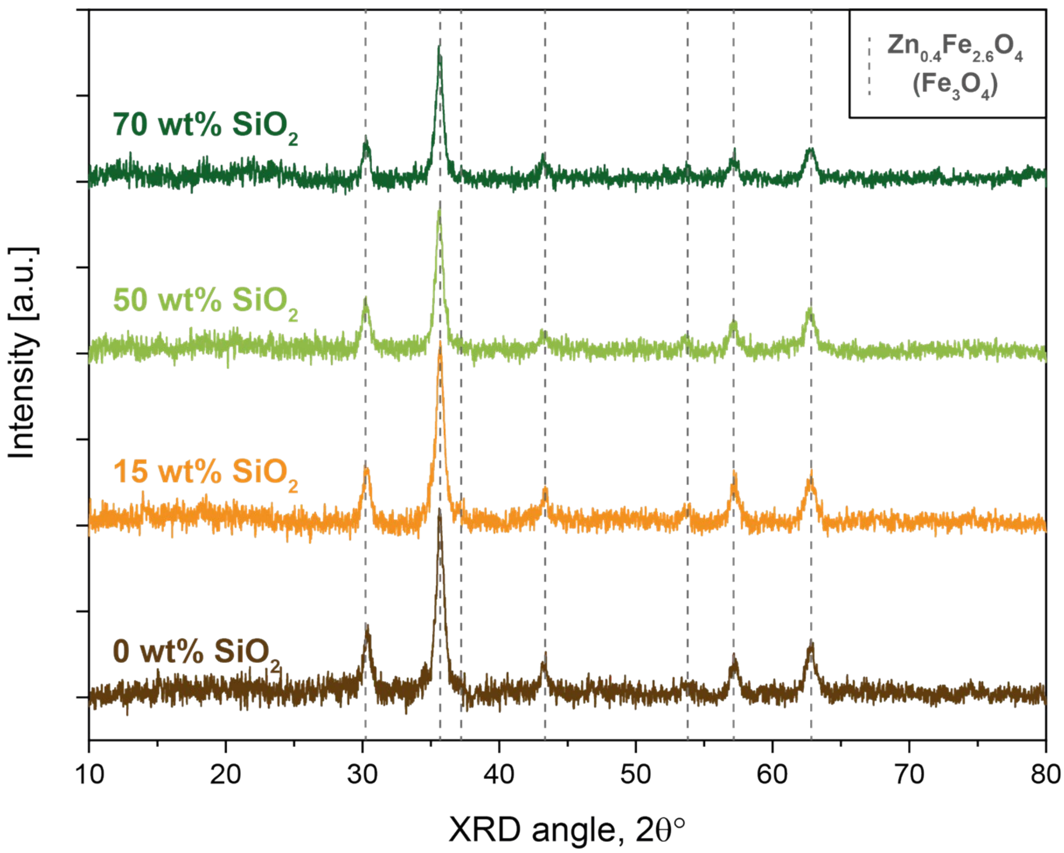

Figure S1. X-ray diffraction patterns of as-prepared $Zn_{0.4}Fe_{2.6}O_4$ nanoparticles with different amounts of SiO_2 coating. All particles show peaks characteristic to Zn-ferrites (Fe_3O_4).

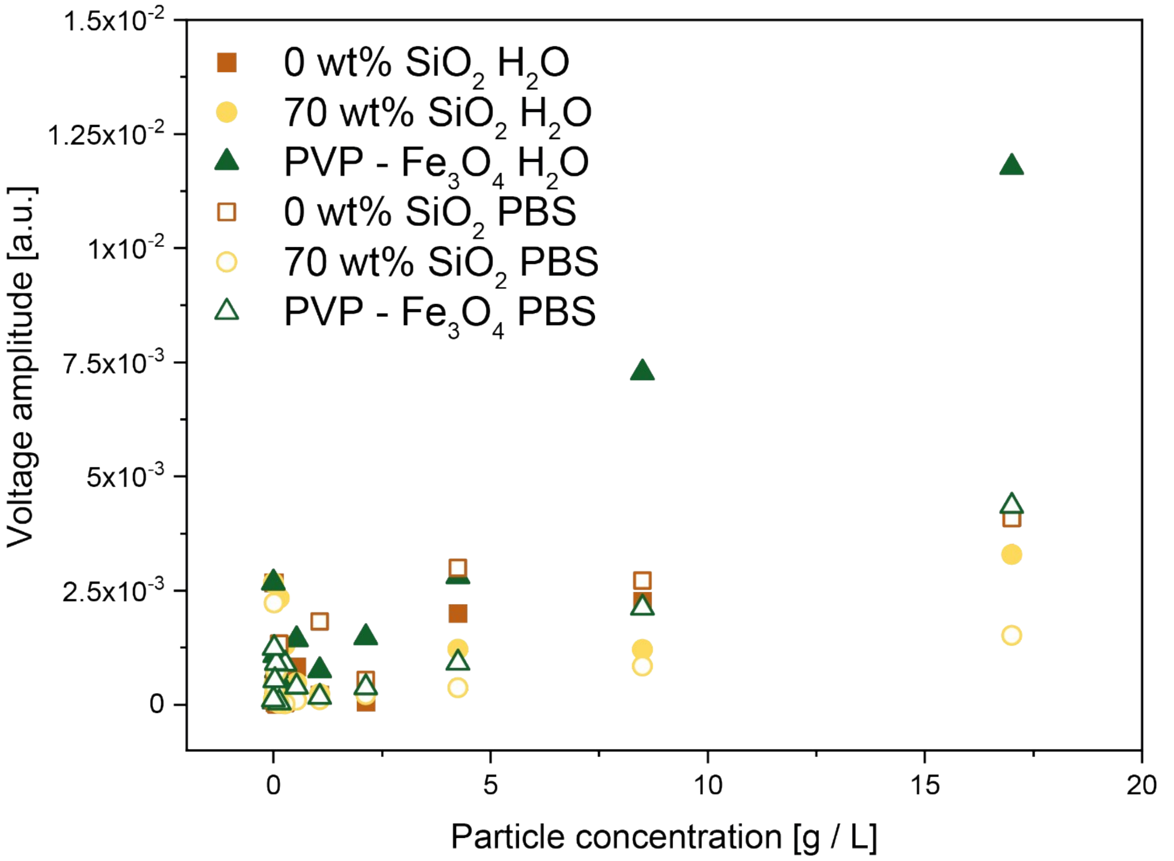

Figure S2. Calibration points for MPS-voltage amplitude for bare and SiO_2-coated (70 wt%) $Zn_{0.4}Fe_{2.6}O_4$, as well as commercial PVP-coated Fe_3O_4, dispersed in H_2O (closed symbols) or PBS (open symbols).

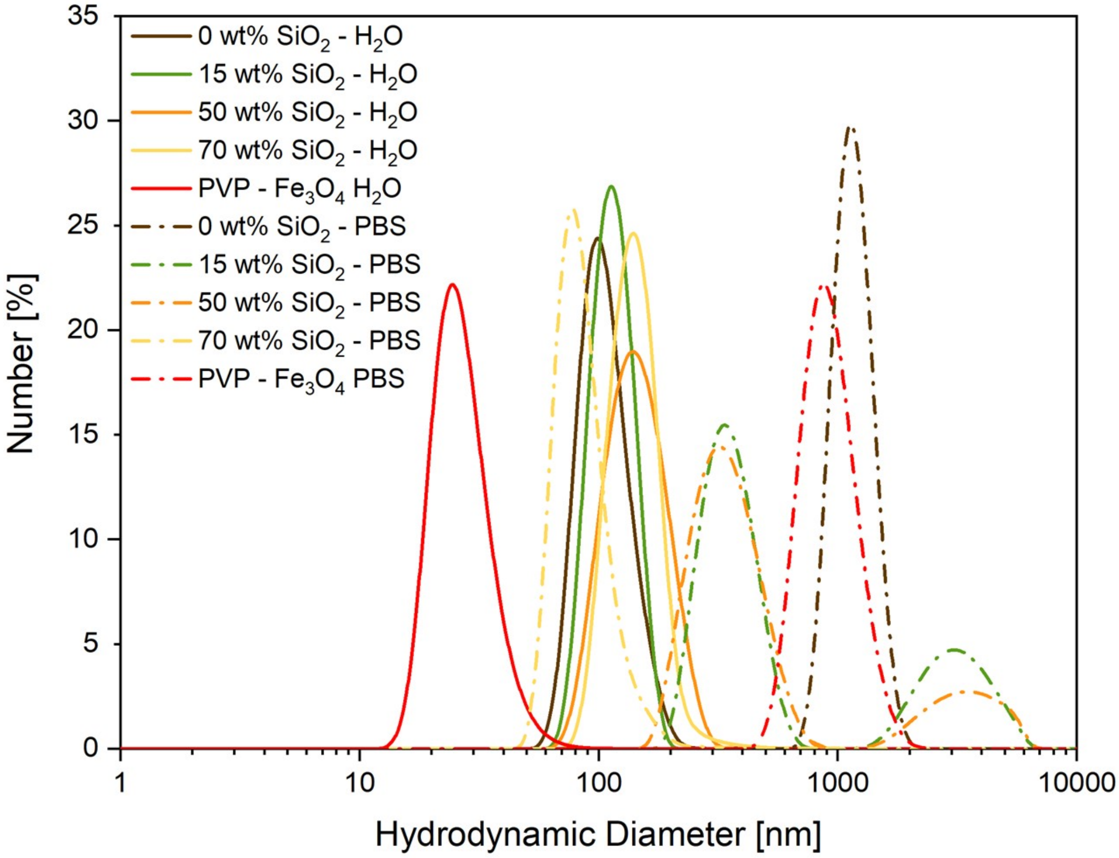

Figure S3. Number-weighted distributions of hydrodynamic diameters bare and SiO_2-coated (15, 50, 70 wt%) $Zn_{0.4}Fe_{2.6}O_4$ as well as of PVP-coated Fe_3O_4 in water and PBS. Measured immediately after sonication at a concentration of 0.1 mg/mL.
